# What Team Science leaders really want: leveraging the knowledge of CTSA-based Team Science leaders to advance the Science of Team Science

**DOI:** 10.3389/fpsyg.2025.1730441

**Published:** 2026-02-10

**Authors:** Betsy Rolland, Kristine M. Glauber, Wayne T. McCormack, Kevin Wooten, Heather J. Risser, Allan R. Brasier

**Affiliations:** 1 Institute for Clinical and Translational Research, University of Wisconsin-Madison, Madison, WI, United States; 2 Michigan Institute for Clinical & Health Research, University of Michigan, Ann Arbor, MI, United States; 3 Clinical and Translational Science Institute, Duke University School of Medicine, Durham, NC, United States; 4 Department of Pathology, Immunology and Laboratory Medicine, Clinical and Translational Science Institute, College of Medicine, University of Florida, Gainesville, FL, United States; 5 College of Business, University of Houston-Clear Lake, Houston, TX, United States; 6 Institute for Translational Sciences, University of Texas Medical Branch, Galveston, TX, United States; 7 Department of Psychiatry and Behavioral Sciences, Feinberg School of Medicine, Northwestern University, Chicago, IL, United States; 8 Department of Medicine, School of Medicine and Public Health, University of Wisconsin-Madison, Madison, WI, United States

**Keywords:** collaboration, cross-CTSA collaboration, exploratory study, interventions, Team Science education, Team Science evaluation

## Abstract

Clinical and Translational Science Award (CTSA) hubs invest substantial resources in facilitating a Team Science approach to research. Despite over 15 years of Science of Team Science research, the field lacks evidence-based interventions that can be implemented by CTSA hubs and others in support of collaborative research. Federal funding agencies increasingly call for team-based research but devote few resources to the development, dissemination, and evaluation of Team Science interventions. This lack of feasible interventions leaves CTSA Team Science leaders and Clinical and Translational Research Teams (CTRTs) themselves with few evidence-based options for improving the impact of team-based research. In this project, a group of CTSA Team Science leaders conducted a pilot study to understand this gap better. First, we engaged in a semi-structured needs assessment to answer two questions: (1) What do CTRTs need to thrive in conducting team-based Clinical and Translational Research, and (2) What do CTSA-based Team Science leaders need to build robust Team Science programs that serve CTRTs? Second, we conducted a landscape assessment to identify existing evidence-informed team-based interventions. Third, we designed and launched a hybrid effectiveness-feasibility project. Here, we report on these three activities. Finally, we call for additional funding to support the development, dissemination, and evaluation of these critical interventions.

## 1 Introduction

Team Science is an essential element of Clinical and Translational Research (CTR), and one of the Translational Science Principles considered key for generating innovation to practice (Austin, 2021; Faupel-Badger et al., 2022). As the Clinical and Translational Science Award (CTSA) hubs work to improve the practice of CTR through a focus on Translational Science, the study of the conduct of CTR (Faupel-Badger et al., 2022), many are seeking evidence-based interventions from the field of the Science of Team Science (SciTS) (National Academies of Sciences, Engineering, and Medicine, 2025) to make teamwork more impactful. Yet, more than 15 years after the first SciTS conference, the field still lacks generalizable, evidence-based interventions – i.e., tools, strategies, and practices that have been rigorously tested, widely disseminated and implemented in service of enhancing Team Science (Rolland et al., 2021b). Existing interventions either allude to or explicitly promise long-term impact such as “more effective teams” or “better science.” Yet the precise pathway to that impact remains nebulous and undefined, as do the specific outcomes implementers or participants can expect. Without explicit attention to the mechanisms of change and expected outcomes, it is impossible to test either efficacy (outcomes under ideal circumstances) or effectiveness (outcomes under real-world conditions) (Singal et al., 2014). Rolland et al. noted that this dearth of evidence-based approaches leaves both research leaders (e.g., CTSA directors, Team Science leaders and staff) and translational researchers struggling to find reliable ways to improve their teamwork (Rolland et al., 2021a).

**FIGURE 1:**
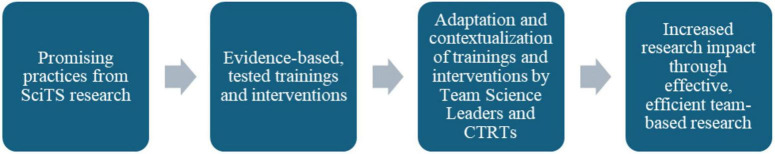
Hypothesized impact of translation of Science of Team Science (SciTS) research into evidence-based trainings and interventions on team-based research. CTRT, Clinical and Translational Research Team.

Previous SciTS research has demonstrated that team-based training leads to enhanced performance, leading to translational impact (National Academies of Sciences, Engineering, and Medicine, 2025). In the realm of CTR, the Translational Science Benefits Model (TSBM) has described impact as comprising some 30 potential benefits across four domains: Clinical, Community, Economic, and Policy (Luke et al., 2018). [An example of a case study of translational team impact of CTRT using the TSBM is described in Brimhall et al. (2025)]. Consequently, we hypothesize that providing teams with “off-the-shelf” tools such as processes, templates, and checklists that can be adapted and customized for their teams and their local contexts will lead to improved team practice. Well designed, rigorously tested, broadly disseminated interventions shown to improve team processes can then increase research impact. (Figure 1: hypothesized impact of interventions). However, as noted above, while many existing interventions purport to advance team science practice, the mechanisms through which individual interventions may operate and their measurable endpoints remain insufficiently defined.

The Institutes and Centers of the National Institutes of Health (NIH), including the National Center for Advancing Translational Sciences (NCATS), regularly call for Team Science approaches and multi-disciplinary research but, to our knowledge, have invested little in developing evidence-based interventions to enhance the conduct of team-based research. In 2008, the National Cancer Institute made such an investment, creating the Team Science Toolkit for sharing knowledge and tools (The National Cancer Institute [NCI], 2024). The Toolkit was used extensively by the Team Science community until its abrupt decommissioning in 2022 (Laursen et al., 2024).

Further, major NIH funding mechanisms (e.g., R-, P-, and U-series awards) do not require the integration of team science interventions or expertise despite the trend for multiple PD/PI award, making it more difficult for the relatively new field of Team Science to gain traction or to test promising interventions. The field’s progress can be compared to that of the field of Implementation Science (Kirchner et al., 2017; Nilsen, 2015), which developed at roughly the same time as SciTS and yet is more advanced in its reach, rigor, and reproducibility. Kim et al. (2023) draw a similar parallel between Implementation Science and the developing field of Translational Science, noting that “[t]he field of implementation science gained traction only when several NIH institutes were willing to fund individual investigator grants on this topic; similar cross-NIH support is needed for [Translational Science] to grow.”

On the one hand, CTSA Team Science leaders know they should be providing interventions to help Clinical and Translational Research Teams (CTRTs) work together more effectively and CTRTs know they should be paying more attention to their team processes. SciTS researchers, on the other hand, want to understand what makes high-functioning teams successful and, subsequently, communicate those findings to the scientific community. Given the applied nature of most SciTS research, we can assume that SciTS researchers are also interested in ensuring their findings are taken up and implemented by science teams. And yet, there remains a gap between the research on and practice of Team Science. The field is missing efforts to translate SciTS findings into actionable, practical strategies for improving teamwork.

In order for the SciTS field to advance and for science teams, especially those in CTR, to conduct high-impact science, we need to fill this gap. One potential path for doing so is by connecting SciTS researchers interested in creating accessible, actionable, evidence-based interventions for advancing teamwork with Team Science leaders and scientific research teams that are willing and able to invest time in implementing and testing those interventions in order to build that evidence base.

Here, we report on a project led by the CTSA Consortium-based Team Science Affinity Group (TSAG) to conduct a needs assessment of TSAG members, identify existing evidence-informed interventions, and connect Team Science leaders with SciTS researchers with interventions that were ready to disseminate and implement in real-world conditions.

## 2 Materials and methods

The authors used a mixed-method design with multiple phases designed to provide adequate sampling of existing CTSA-based Team Science programs.

### 2.1 Phase 1–needs assessment

In Phase 1, several of the authors (BR, KG, WM, KW) conducted a structured needs assessment with TSAG participants. The TSAG consists of CTSA Team Science leaders who meet monthly to discuss Team Science in CTR. (In March 2024, the TSAG restructured as the TSAG Research and Ideas Showcase and the Team Science Professionals Special Interest Group of the ACTS.) In two such TSAG meetings, we implemented a process through which participants identified key requirements for delivering team-based interventions at their CTSAs. Through this needs assessment, we sought to answer two questions: (1) What do CTRTs need to thrive in conducting team-based CTR, and (2) What do CTSA-based Team Science leaders need to build robust Team Science programs that serve CTRTs?

Our needs assessment was structured around seven key SciTS research gaps highlighted in Hall et al. (2018):

1. Impact and productivity of Team Science
2. Team configuration challenges
3. Multiteam systems
4. Leadership in Team Science
5. Training for Team Science
6. Technology
7. The need for more sophisticated study designs and methods

We split these seven topics over 2 months (February and March 2022). Thirty-five TSAG members participated in the first discussion and 25 TSAG members, in the second. Topics were discussed in breakout groups, with participants self-selecting which topic they wanted to discuss. While we did not record the number of participants in each breakout, participation was relatively even across topics. Each breakout was led by a facilitator who led participants through the following questions for each topic:

- What are you, as a CTSA Team Science leader, struggling with in this area?
- What do you want to know to help you run your Team Science program better and help you better support your CTRTs?
- What research questions would you like to ask about facilitating CTRTs?

Responses were collected in a set of Google docs, with both participants and facilitators contributing to capturing the conversation. After each of the two TSAG meetings, an email was sent to the TSAG email list with links to the docs and an invitation to those not in attendance to edit or annotate. We also encouraged meeting participants to review all the conversations, not just those in which they had participated.

**FIGURE 2:**
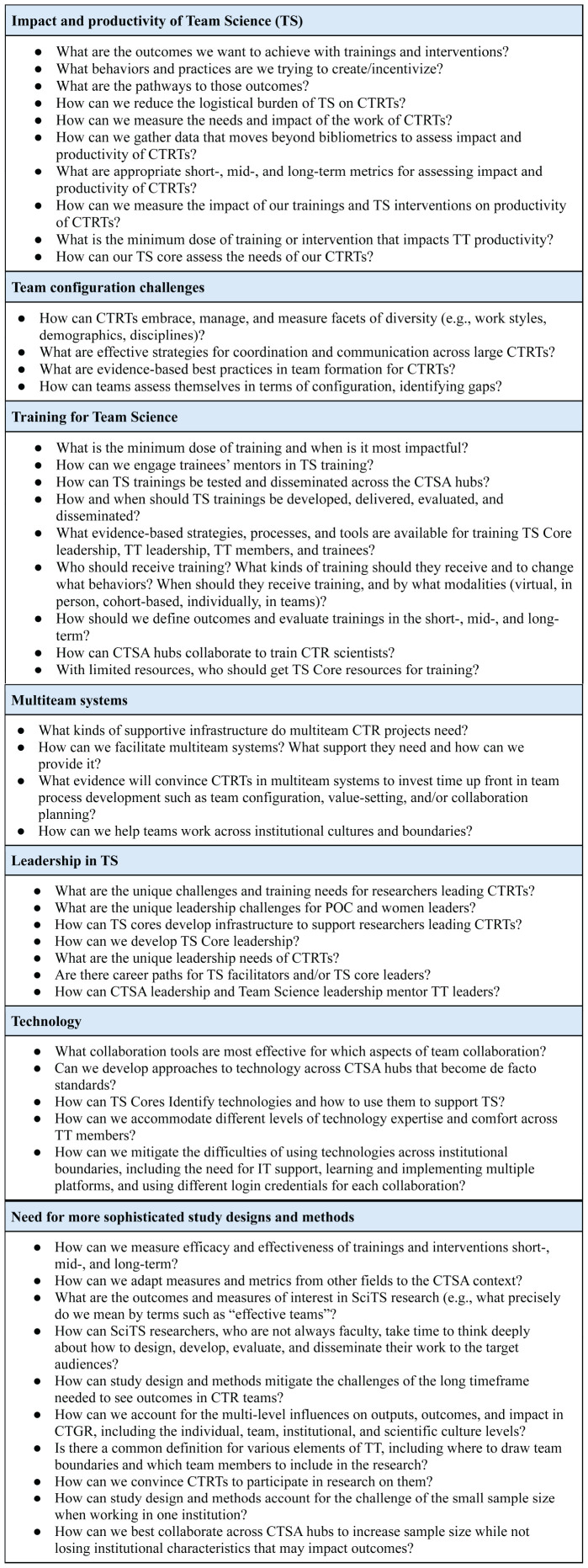
Opportunities, challenges, and potential research questions identified by Team Science Affinity Group (TSAG) members at February and March 2022 sessions. Topics from Hall et al. (2018).

After the two sessions, the project leadership (BR, KG, WM, KW) consolidated the raw data. First, BR, a trained qualitative researcher, conducted an initial analysis of the data, consolidating similar statements, doing light editing for clarity, removing any institution- or participant-specific details in the data, and grouping statements into themes for further summarization. This initial analysis was then reviewed and discussed with the leadership team, assessing alternative interpretations of the data and coming to an agreement on the final summary. All statements were reframed as questions for consistency and consolidated into Figure 2.

The investigators will make de-identified primary data available to qualified investigators upon request.

### 2.2 Phase 2–landscape assessment

As the group’s desire for tools, strategies, and practices they could implement with CTRTs became clear, the TSAG committee developed criteria to identify viable team-based interventions that could potentially be disseminated and implemented at the CTSA hubs. First, the intervention must meet the definition of intervention in Rolland et al.: “one strategy or a set of strategies that have been systematically implemented with a team and evaluated, with demonstrated impact” (Rolland et al., 2021b). Second, we sought interventions that had been implemented and evaluated at least once. Third, an intervention must have been shown to improve the practice of Team Science in at least one local environment. Given the still-developing state of the SciTS field, “improve the practice” was defined loosely as having some evidence of effectiveness, even as minimal as “participant satisfaction.” The TSAG committee agreed to be as generous as possible in our assessment of “evidence of effectiveness” and “demonstrated impact,” operationalizing these constructs as having conducted any evaluation of their implementation. As noted, to our knowledge, at the time, no Team Science interventions had been rigorously tested in real-world conditions, and our inclusion criteria and our application of them needed to reflect that reality.

Our landscape assessment relied on two key sources to identify potential interventions: (1) Team Science experts in the SciTS community and (2) the SciTS literature. First, we queried Team Science experts through the TSAG membership, the NIH-hosted SciTS listserv, and the INSciTS board of directors. Second, we worked with the UW-Madison library to query the literature to identify any additional Team Science interventions. Our assessment focused specifically on interventions designed for and tested with scientists or science teams. We did not consider team-based interventions that were targeted toward generic teams or toward teams outside of scientific research.

### 2.3 Phase 3–connecting CTSA-based Team Science leaders and intervention developers

List of interventions in hand, TSAG leadership explored options for connecting Team Science leaders and intervention developers through what we called “The TSAG Pilot Project.” The goal of this project was to invite intervention developers to train CTSA-based Team Science leaders in implementing their interventions with CTRTs served by the CTSA, while gathering data on the effectiveness of the intervention itself in real-world conditions. We modeled this phase on implementation science approaches such as hybrid effectiveness-implementation trials (Curran et al., 2012).

The developers of the five identified interventions were invited to participate in this project. Participation required delivery of an implementation training session [i.e., a train-the-trainer model; e.g., (Pfund et al., 2015)], materials, and minimal ongoing support; and agreement to collect a modest set of common metrics. One program (The Toolbox Dialogue Initiative) operates on a fee-for-service model, which was made clear to potential participants in the overview. All other interventions were free for CTSA hubs to implement.

**FIGURE 3:**
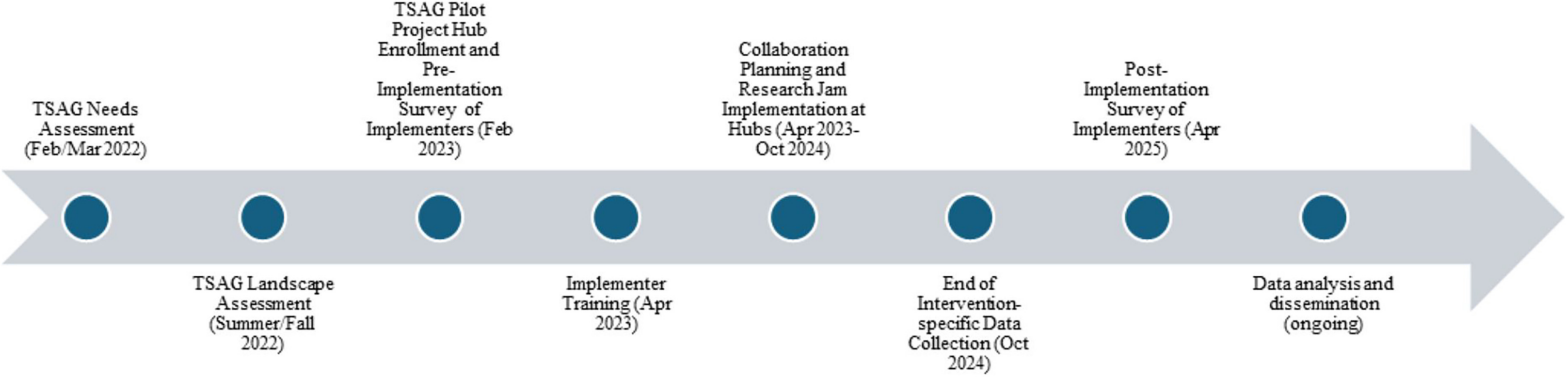
Team Science Affinity Group (TSAG) pilot project activities timeline.

The TSAG Pilot Project Committee (see Section “Acknowledgments”) devised implementation-focused evaluation criteria which included repeated measures to be collected from all implementers (i.e., CTSA Team Science leaders) to assess the feasibility and usability of implementing the interventions (pre- and post-implementation period) (see Figure 3 for project activities timeline). We focused on feasibility and usability as key constructs from the field of Implementation Science, with more in-depth assessments of effectiveness of the interventions themselves to come in subsequent studies. We adapted our feasibility question from Weiner et al. (2017) and our usability questions from the System Usability Scale (Gallavin, 2024) (see Supplementary materials for feasibility and usability questions). Each intervention was also encouraged to deploy its own intervention-specific evaluation directly with implementing sites. For example, Collaboration Planning and Research Jams had existing evaluation criteria they deployed during implementation. These intervention-specific evaluation criteria will be discussed in detail in a future publication.

The project was launched in February 2023, with a website and marketing materials distributed to Team Science and CTSA-focused listservs. After an information session, we opened the application process to any funded CTSA hub. Each hub indicated which intervention(s) they wanted to implement based on local priorities and needs. We requested that implementers provide a Letter of Commitment from their leadership specifying the allocated staff and effort, to ensure that leadership was aware of the commitment and to minimize sites dropping out because they had not committed sufficient resources. Notably, we did not offer any funding to either intervention developers or to potential CTSA implementation sites. All work would need to be done using their existing resources. Coordination efforts were supported by the University of Wisconsin-Madison Institute for Clinical and Translational Research (UW-ICTR). The project officially closed as of October 1, 2024.

## 3 Results

### 3.1 Phase 1–needs assessment

The needs assessment provided data to answer two questions: (1) What do CTRTs need to thrive in conducting team-based CTR, and (2) What do CTSA-based Team Science leaders need to build robust Team Science programs that serve CTRTs? A summary of responses to both questions is shown in Figure 2.

#### 3.1.1 What do CTRTs need to thrive in conducting team-based CTR?

There was a general consensus among the Team Science leaders that one of the main reasons CTRTs rarely engaged with Team Science interventions was simply a lack of awareness, followed closely by a lack of time to invest in their team processes. In their experience, CTRTs were generally unaware of specific resources offered by the CTSA to facilitate Team Science, specifically, and of the existence of SciTS as a field of study, more generally. As such, more targeted education for CTRTs was a key need. Additional areas of identified need included:

- Infrastructural support for collaboration in the form of available experts or easy-to-use guidelines for forming, building, leading, managing, evaluating, and sunsetting productive, diverse, satisfied, and successful teams
- Easy-to-implement interventions provided by their CTSA that will make conducting high-impact CTR easier and can scale to large, distributed and multi-team system projects
- Effective, practical trainings for CTRT leaders and members that lead to increased self-efficacy, greater productivity, successful projects, and team satisfaction
- Help choosing technologies that support teamwork (e.g., communication and coordination platforms)
- Understanding that their team processes impact scientific outcomes and there are interventions that can enhance their processes.

#### 3.1.2 What do CTSA-based Team Science leaders need to build robust Team Science programs that serve CTRTs?

Without formal training programs or clear career paths for Team Science leaders, many are relatively new to the field and still building their knowledge of SciTS. Further, few resources exist for building Team Science programs. As such, TSAG members felt unsure of how to build CTSA-supported Team Science units that truly served the needs of their constituents. Additional areas of need included:

- Evidence-based, actionable, accessible, active, affordable interventions (tools, strategies, and practices) that are ready to disseminate to CTRTs, have defined outcomes, and don’t require large investments of time or money from the frequently under-resourced Team Science facilitators or overstretched CTRTs
- Simple short-, mid-, and long-term metrics to assess the intervention process and outcomes that can be used to show value and return on investment
- Outcomes data to show institutional leaders, funding agencies, and CTRT leaders that investing resources in these interventions is impactful
- Intervention evaluation data that can be used to easily assess and compare interventions and choose what is right for a given context, based on their CTSA hub’s priorities and desired outcomes, as well as to recommend to CTRTs based on their specific needs
- Fundamental training in Team Science and in facilitation, including team needs assessment and intervention delivery, to support CTRTs
- A forum for connecting with the SciTS community that leads to bi-directional engagement (i.e., be the “community” in community-engaged research)

In short, Team Science leaders wanted evidence-based tools, strategies, and practices they could deliver to CTRTs and the training to do so.

### 3.2 Phase 2–landscape assessment

**FIGURE 4:**
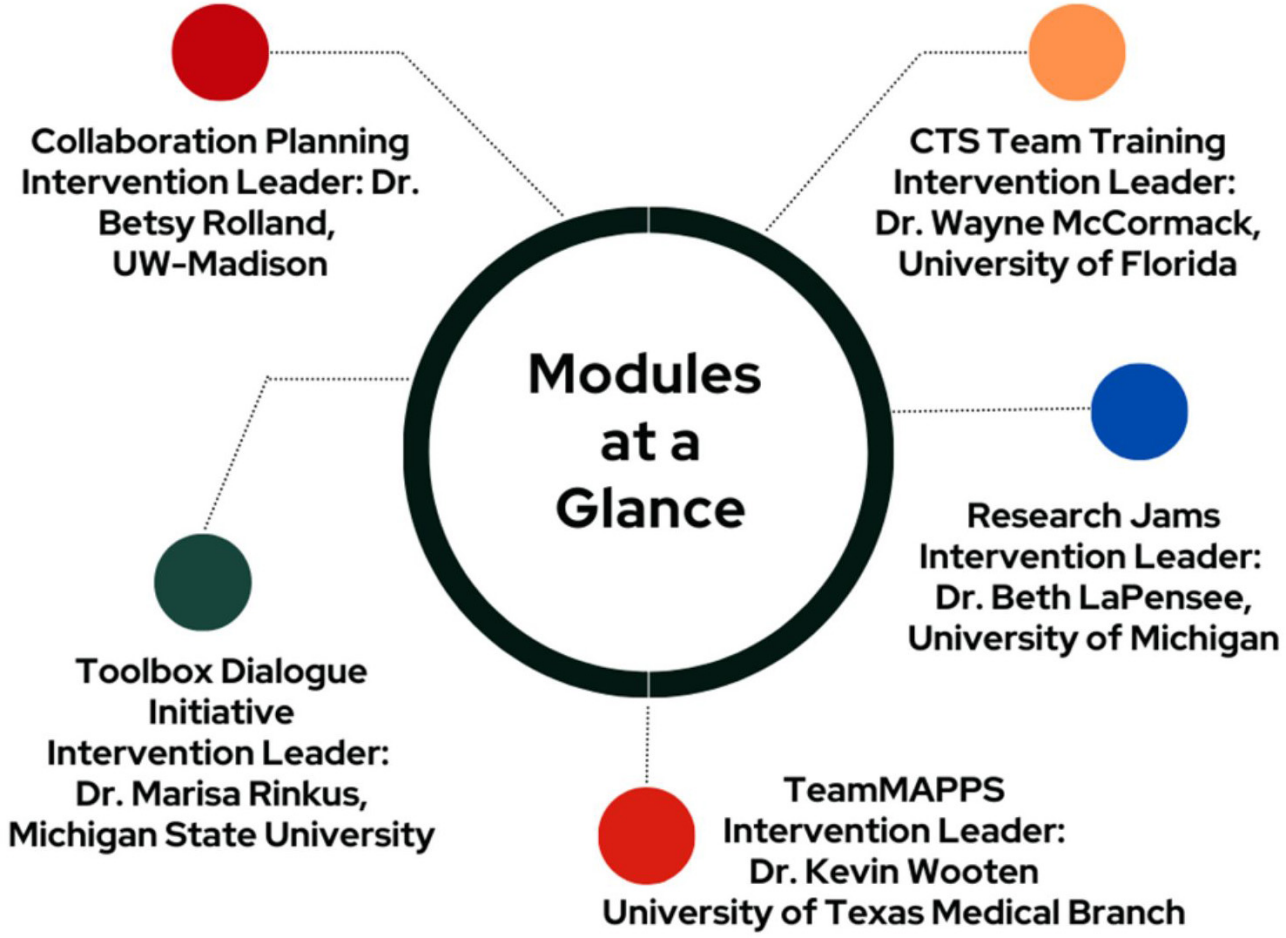
Team Science Affinity Group (TSAG) pilot study interventions. CTS, Clinical and Translational Science; TeamMAPPS, Team Methods to Advance Processes and Performance in Science (please see Supplementary materials for more details about each intervention).

Our landscape assessment identified five interventions that met our criteria; these five interventions were the only Team Science interventions the group was able to identify (Figure 4). Notably, all five were already being disseminated, adding urgency to our exploration of their effectiveness with CTRTs in Phase 3. Each intervention is described briefly here; more details are available in the Supplementary materials or via referenced publications.

- Collaboration Planning (Rolland et al., 2020, 2025): Collaboration Planning is a 90-min intervention for translational teams to help them think through their approach to collaboration, proactively addressing the areas that most frequently cause conflict in teams, including authorship, communications, and project management.
- CTS Team Training (McCormack and Levites Strekalova, 2021): Clinical and Translational Science (CTS) Teams are pairs of Ph.D. or dual-degree trainees drawn from distinct disciplines and colleges, whose collaborative “team-specific aims” are embedded into their dissertation research and supported by cross-disciplinary mentoring and a team science curriculum.
- Research Jams (LaPensee et al., 2021, 2024): Research Jams are facilitated sessions designed to assist diverse groups in surfacing cutting-edge research ideas, building collaborations, creating shared research agendas, and developing strategic action plans.
- TeamMAPPS (Bisbey et al., 2021; Molldrem et al., 2025): TeamMAPPS (Team Methods to Advance Processes and Performance in Science) is a behavioral skills training program specifically developed to increase the collaboration and effectiveness of scientific teams and scientific team members.
- Toolbox Dialogue Initiative (Graham et al., 2020; Rinkus et al., 2021): Developed by the Toolbox Dialogue Initiative (TDI), the Toolbox dialogue method is an evidence-informed facilitation approach that surfaces implicit assumptions and diverse perspectives in complex, cross-disciplinary research projects for joint consideration and coordination.

### 3.3 Phase 3–connecting CTSA-based Team Science leaders and intervention developers

**FIGURE 5:**
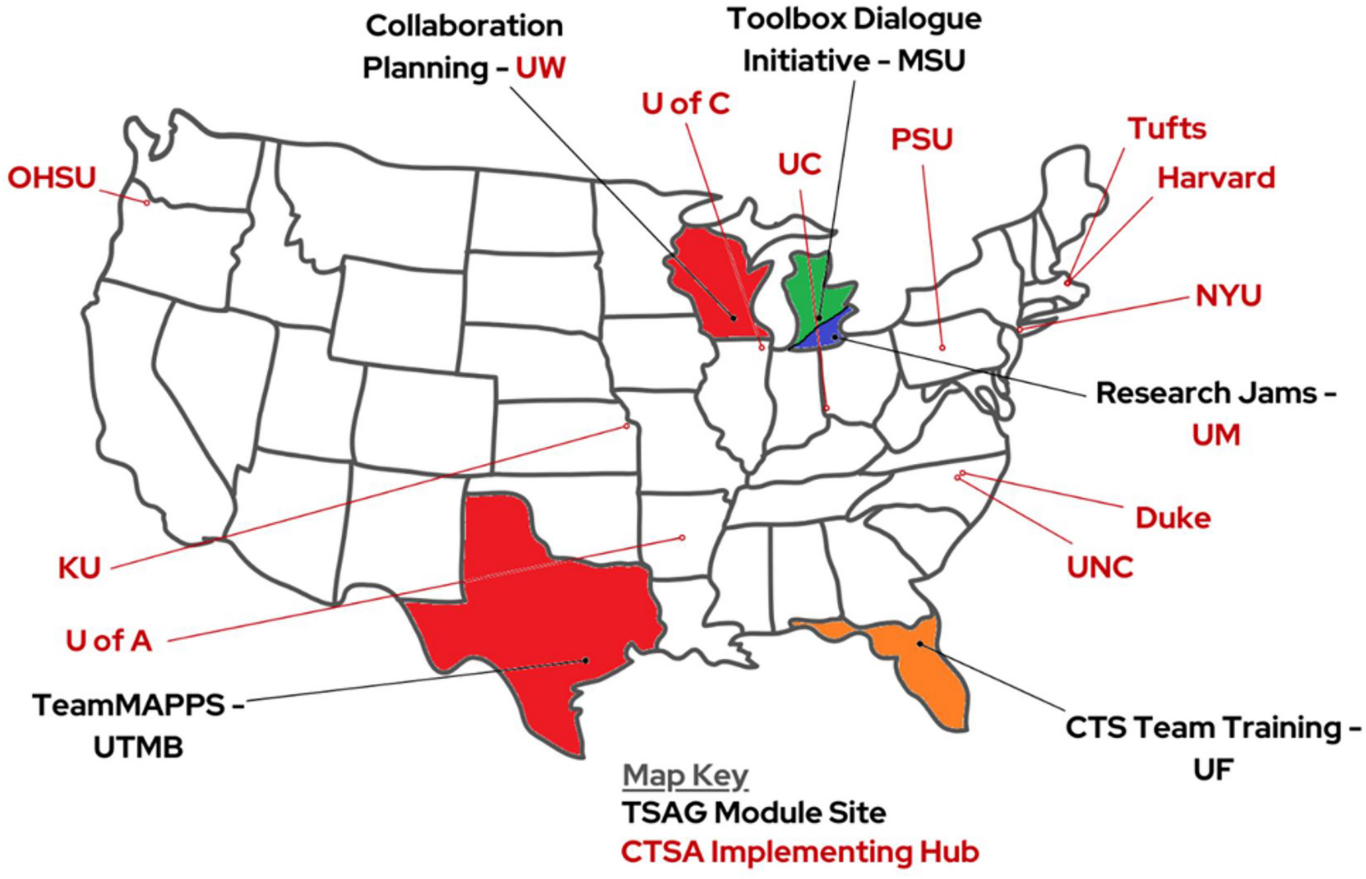
Map of TSAG intervention and implementation participants.

The developers of all five of the identified interventions agreed to participate in this project (Figure 4). Thirteen CTSA hubs applied to participate (Figure 5), representing approximately 20% of then-funded CTSA hubs. Enrollment data on hub size indicate that six of the hubs were “Small” (<$4.5M), two were “Medium” ($4.5 < $6M), and five were “Large” (>$6M). Several additional sites told us they would be interested in participating in the future but needed a longer lead time to get institutional buy-in. Application data are summarized in Table 1. Three interventions received sufficient enrollment to proceed, as defined by each intervention developer based on training and support resources: Collaboration Planning (CP), Research Jams (RJs), and TeamMAPPS. While we did not collect formal data on Team Science leaders’ reasons for which intervention(s) they chose, anecdotal data from conversations indicate that CP, RJs, and TeamMAPPS were viewed as more accessible and requiring a lower resource investment than some of the other offered interventions.

**TABLE 1:** Team Science Affinity Group (TSAG) pilot study enrollment data.

Intervention	# Hubs expressing interest in implementing
Collaboration Planning	12
Research Jams	4
TeamMAPPS	2
CTS teams training	1
Toolbox dialogue	1

Further implementation details, as well as outcome and evaluation data from the implementation of this study will be shared in future publications.

## 4 Discussion

The conduct of CTR requires a team-based approach, and CTSAs are increasingly seeking to offer accessible, evidence-based resources to support CTRTs in improving their teamwork and team processes. In the work described here, we share the results of a structured needs assessment that identified the needs of CTSA-based Team Science leaders and the teams they support, as well as our findings from a landscape assessment of existing team-based interventions that have the potential to meet those needs. Finally, we share how CTSA-based Team Science leaders and intervention developers embraced a project to connect them with one another, with outcomes and evaluation results to come in future publications. While previous work has identified what science teams need to improve their team processes and increase the efficiency and effectiveness of their work, little research has identified how those needs can be met through facilitation or what support such Team Science facilitators might themselves need (National Academies of Sciences, Engineering, and Medicine, 2025). As such, here we essentially describe a research agenda for SciTS researchers interested in addressing this gap.

The 2013 Institute of Medicine review of the CTSA program identified that CTSA programs were uniquely positioned to promote and disseminate innovations in Team Science that promote its adoption and effectiveness (National Research Council, 2015), making them an ideal testbed for SciTS researchers. Despite substantial work in promoting CTR, this TSAG study is the first, to our knowledge, to examine barriers and facilitators for effective adoption of team science interventions across CTSA organizations. Our study includes representation from small and large Hubs, whose size is reflective of aggregate extramural NIH funding received by the institution, as well as size and diversity of its faculty. Consequently, we expect that these findings likely will be generalizable to the larger CTSA network and can inform SciTS research around translational of findings into practice, as well as serve as common ground for bringing together Team Science leaders and SciTS researchers.

Irrespective of the size of the CTSA Hub, the Team Science interventions most likely to be of interest were those that demanded fewer resources from either the team science professionals or the CTRTs involved, indicating a preference for lightweight training interventions. Outside the scope of this study, this information can be used to inform future strategies for structuring just-in-time team-based learning interventions that will be sustainably adopted across the consortium. Additionally, despite the spectrum of Hub composition, we find that complex interventions requiring dedicated time and resources from the team science core to implement the training are seen as less valuable.

Our results indicate that, despite more than 15 years of progress in SciTS research, a gap remains between what we know about how science teams improve their teamwork and how we directly support that improvement. CTSA-based Team Science leaders did not feel they had the resources (in terms of either knowledge or bandwidth) to adequately support CTRTs. Generally new to the field and lacking any formal training in SciTS, they did not feel prepared to take key SciTS findings and translate them into the evidence-based (or even evidence-informed) practices, tools, and strategies that would help their CTRTs improve their teamwork. They were unsure of how to navigate the landscape of existing SciTS knowledge to create interventions they could easily implement with their teams.

The interest we saw in piloting this approach of connecting intervention developers (all SciTS researchers) with Team Science leaders (primarily practitioners) indicates that both sides of this gap have tremendous levels of interest in connecting. Team Science leaders are eager to serve as testbeds, while intervention developers are eager to test their interventions in real-world conditions with evaluation criteria that move beyond simple satisfaction metrics.

In part, this implementation challenge indicates a gap in the research-to-practice pipeline in SciTS overall. Few in the SciTS research community have experience in both SciTS research (i.e., conducting primary research to identify factors that influence team effectiveness) and practice (i.e., working as part of a large scientific research team), which can make it challenging to translate their own findings and those of the community at large into accessible, easy-to-implement interventions for science teams. Even fewer are trained in the approaches of Implementation Science or User-Centered Design that might provide the frameworks and approaches to bridge the gap.

To advance the development of truly evidence-based interventions (rather than simply evidence-informed), a stepwise approach is needed. First, we must assess whether these interventions are suitable for broad dissemination and implementation, given that most were developed within a particular organizational context, and substantial evidence indicates that team context significantly impacts the effectiveness of trainings and interventions (Rolland et al., 2021c). Second, we must begin the work of articulating clear logic models and establishing robust measures that allow us to evaluate their actual effectiveness. The project described here is a first step toward these goals and indicates a direction that might assist the SciTS field in increasing its own reach and impact.

Our needs assessment also revealed the importance of metrics and standardized evaluation criteria for Team Science leaders. Team Science leaders want to choose and invest in delivering interventions they believe most likely to have impact in their local contexts while demonstrating the value of those efforts to their CTSA leaders. Yet, to our knowledge, no such commonly accepted, rigorously developed and validated metric exists that can assess outcomes such as “effective” or “successful” scientific work, raising the question, What kinds of organizational and process metrics are required to demonstrate optimal teamwork? The SciTS field would benefit from increased attention to defining these potential outcomes and developing validated measures that are flexible and adaptable enough to be used by intervention developers, Team Science programs, and the teams themselves.

Further, both SciTS researchers and Team Science leaders would benefit from investment in facilitated co-creation processes that result in user-centered interventions. Without the participation of Team Science leaders and CTRTs themselves, it is nearly impossible for intervention developers to disseminate and test their interventions. In our own work, we have seen that it can be a struggle to convince busy researchers and their teams to participate as research participants. Indeed, recent work showed a discouraging 1.8% return rate for team recruitment, asking the question, “How can the Science of Team Science field advance without the input of the very teams we seek to support?” (Venkatesh and Rolland, 2025) Without a deeper understanding of how to enroll science teams in our research and of what makes research participation compelling and worthwhile for teams, intervention developers will struggle to create the evidence base for their work.

Our work in promoting team science trainings and interventions addresses availability of education and team facilitation, two important organizational factors supporting innovative Team Science within a complex university environment. Other organizational factors include Departmental/Center organization structures, academic cultures of collaboration, incentive/reward structures and others; these have been previously reviewed and will not be recited here (Klein, 2010; National Research Council, 2015). Further in-depth studies of the organizational factors in which Team Science leaders operate within CTSA Hubs will require further study.

Finally, all of this work requires funding. While the TSAG Pilot Project was conducted with no additional funding beyond the centralized marketing and data collection support provided by UW-ICTR, the “all-volunteer” approach is suboptimal. First, such an approach guarantees that only the most well resourced Team Science programs can participate and benefit from providing enhanced support to CTRTs. Second, it fails to incentivize intervention developers to test their interventions rigorously or disseminate them widely while collecting evaluation data about their performance in real-world conditions.

### 4.1 Limitations

There are several limitations to this work. First, the work took place with Team Science leaders at CTSAs, who may not be representative of Team Science leaders in other organizations seeking to encourage collaborative Team Science. Further, the Team Science leaders participating in the TSAG meetings may not have been representative of those across the CTSA hubs who did not participate. Second, this project was implemented with no additional funding, which limited both the scope of the project and the ability of some Team Science leaders to participate. As such, we view this project as a pilot study rather than a fully-fledged research project.

Little research has focused on this population – Team Science leaders seeking to encourage and/or facilitate collaborative Team Science – making it difficult to assess how different or similar CTSA-based Team Science leaders or TSAG participants may be in relation to those at other organizations. As such, it is difficult to know how our participant sample may have biased our results. In future work, we hope to engage with additional Team Science facilitators, including those at lower-resourced CTSA hubs, NCI-designated cancer centers, administrative directors of large research initiatives, and others seeking to encourage and support collaborative research.

## 5 Conclusion

In order to extend its reach and advance its mission of helping science teams operate more effectively and efficiently, the SciTS field must invest in translating its key findings into accessible, actionable interventions that teams, including CTRTs, can implement. That said, SciTS researchers cannot do this alone. Funding agencies must also invest in funding SciTS research and its translation and implementation. Organizations such as CTSAs, NCI-designated cancer centers, and large NIH-funded consortia must engage with SciTS researchers and Team Science experts to identify interventions that might help science teams improve their teamwork and serve as participants in testing the interventions.

CTSAs, in particular, need tools that help CTRTs improve teamwork and can increase teams’ potential to improve human health in order to fulfill their mission. This focus on collaborative, team-based research means the CTSAs have the potential to serve as an ideal testbed for intervention developers who want to collect implementation and effectiveness data in real-world conditions. Team Science leaders, such as those the CTSAs frequently employ, can serve as liaisons between these two constituencies. This pilot project is a first step toward collecting real-world data that leaders can use to better support CTRTs at their Hubs and facilitate high-impact science. The project was received by both intervention developers and CTSA hubs with enthusiasm and is starting to fill a need for off-the-shelf interventions that CTSA Team Science leaders can implement to help their teams be more successful.

We have been impressed and gratified by the willingness of CTSA hubs to invest resources in implementing Team Science interventions and contributing to the evidence base and that of intervention developers investing resources in creating dissemination packages that can be implemented by those Team Science leaders. This energy is indicative of a real need that is going unfulfilled due to the lack of funding to develop, evaluate, and disseminate team-based interventions. Without such funding, intervention developers find it difficult to rigorously establish the evidence base for the effectiveness of their interventions, which consequently leaves Team Science leaders and CTRTs without the tools they need to conduct high-impact CTR.

## Data Availability

The datasets presented in this article are not readily available because they may contain identifying details. A deidentified data set may be made available to qualified investigators by emailing berollan@med.umich.edu.
