# Supplementary material for: What Team Science leaders really want: leveraging the knowledge of CTSA-based Team Science leaders to advance the Science of Team Science
Source: Front Psychol. 2026 Feb 10;16:1730441. doi: 10.3389/fpsyg.2025.1730441 (PMC12929508; doi:10.3389/fpsyg.2025.1730441)
Supplement: Supplementary file 1 [file Table_1.docx]

**Supplementary Materials**

**Feasibility and Usability Questions from Pre- and Post-Implementation Surveys**

**These questions were asked for each intervention the Hub selected.** **Here, we present the questions as displayed for Collaboration Planning in the pre-implementation survey. Questions were altered for the post-implementation survey to reflect the past tense (e.g., from “Collaboration Planning will be feasible” to “Collaboration Planning was feasible”). All questions used a 5-point Likert scale from Strongly Disagree to Strongly Agree.**

1. Feasibility: An evidence-based practice can be judged feasible if a task or an action can be performed relatively easily or conveniently given existing resources (e.g., effort, time, and money) and circumstances (e.g., timing or sociopolitical will). [Adapted from [1]]
   1. Collaboration Planning will be feasible to implement at my organization.
2. Usability: An evidence-based practice can be judged usable if it helps users achieve specified goals with effectiveness, efficiency, and satisfaction. [Adapted from [2]]
   1. I anticipate I would like to use Collaboration Planning frequently.
   2. I anticipate Collaboration Planning will be unnecessarily complex.
   3. I anticipate Collaboration Planning will be easy to use.
   4. I anticipate that I will need the support of a technical person to be able to use Collaboration Planning.
   5. I anticipate the various functions in Collaboration Planning will be well integrated.
   6. I anticipate there will be too much inconsistency in Collaboration Planning.
   7. I anticipate that most people will learn to use Collaboration Planning very quickly.
   8. I anticipate Collaboration Planning will be very cumbersome to use.
   9. I anticipate feeling very confident about my ability to use Collaboration Planning.
   10. I need to learn a lot of things before I can get going with Collaboration Planning.

**Study Intervention Overviews Presented to Team Science Leaders**

**1. Collaboration Planning [3,4]**

**Project Leader:** Dr. Betsy Rolland

**Project Institution:** University of Wisconsin-Madison

**Module Description:**

Collaboration Planning is a 90-minute intervention for translational teams to help them think through their approach to collaboration, proactively addressing the areas that most frequently cause conflict in teams, including authorship, communications, and project management. Originally designed by team-science experts at the NIH and NSF drawing upon decades of research on teams and collaboration, a collaboration plan may be useful in the following ways:

- To help your team create strong team processes from the beginning, which has been shown to result in improved scientific outcomes
- As a template for writing a multi-PI plan
- To address team-science-specific review criteria in future grant applications
- Skill-building for research staff and early-stage investigators in team leadership and collaboration

UW–ICTR’s Collaboration Planning intervention has been developed and tested with more than 40 teams at UW–Madison. Over the course of this testing, we have honed the content to meet the needs of teams at various stages of development.

**2. CTS Team Training [5]**

**Project Leader:** Dr. Wayne T. McCormack

**Project Institution:** University of Florida

**Module Description:**

The CTS Teams intervention is described in some detail in this publication, which is required reading for considering this training intervention: McCormack WT, Levites Strekalova YA. 2021. CTS Teams: A New Model for Translational Team Training and Team Science Intervention. Journal of Clinical & Translational Science 5(1), E183

For the purpose of this TSAG training/intervention pilot study, it is hoped that partner programs will include both components of the CTS Team training model: didactic (a one-semester Team Science course) and experiential (TL1/T32 support of cross-disciplinary teams [pairs] of trainees). The CTS Team training model was developed and implemented at the University of Florida Clinical & Translational Institute by Wayne T. McCormack, PhD. Since 2016, TL1 trainees have applied for training grant support with a research partner (PhD and or MD-PhD students) who is earning their PhD degree in a different discipline, graduate program, and college. Students apply typically in their second year of PhD studies, before they defend their PhD dissertation proposals. They propose “team specific aims” that involve collaborative research, require cross-disciplinary collaboration to accomplish, and become embedded in their respective PhD dissertation research projects. Teams are typically supported for two years, beginning in the Fall semester. We use a variation of this team training model in a UF T32-funded cancer biology training program with both predoctoral and postdoctoral trainees.

Didactic training in team science & collaboration skills is provided by a one-credit semester-long “Team Science” course (one 50-minute class session per week, 15 weeks). The course syllabus may be viewed here: https://mccormacklab.pathology.ufl.edu/teaching/gms-6945-team-science/. Ideally partner programs would implement the entire CTS Team training model, however, we are open to the possibility of some programs only adapting the “Team Science” course (didactic training) to their program, to compare learning outcomes with the full program. Resources available to support implementation of the course include PowerPoint files, recorded lectures, lecture transcripts, and (under development) facilitator guides for each session. It is important to note that this course involves multiple homework assignments that trainees should work on in their teams and/or with consultation with other lab members and/or their mentors. Homework assignments allow trainees to immediately apply team science principles to their own research.

The experiential element of this program is the two-year TL1/T32-funded research experience. In practice this experience actually extends beyond the two-year funding period until students complete their PhD degree. Team specific aims are embedded into dissertation research projects, and are sometimes not completed within two years. Outcomes from the first several cohorts include improved self-efficacy for clinical research skills, a significant increase in the frequency of participation in cross-disciplinary collaborative activities by both trainees and mentors, and intention by co-mentors (most of whom had not previously collaborated with each other) to continue collaborating beyond the period of training grant support. CTS Teams provide a unique environment in which to study the dynamics of cross-disciplinary team mentoring. Mentor/mentee training materials and team mentoring assessment tools are under development, and will be piloted as part of this project.

**3. Research Jams [6,7]**

**Project Leader:** Dr. Beth LaPensee

**Project Institution:** University of Michigan

**Module Description:**

Research Jams are facilitated sessions designed to assist diverse groups in surfacing cutting-edge research ideas, building collaborations, creating shared research agendas, and developing strategic action plans. We have developed multiple Research Jams frameworks that have different purposes and intended outcomes. We are best positioned to disseminate our Ideation Jam – a framework ideal for groups who want to mobilize toward addressing a complex or ‘wicked’ research problem. A half day session, often with participants who are meeting for the first time, uses design thinking methods to move through phases of blue-sky thinking, pattern building, prioritization, mapping interest, and action planning. Ideation Jams foster a psychologically safe environment for co-creation and utilize a range of activities that promote individual ideation and collaborative exploration. We distill all Ideation Jam outputs into a report or virtual whiteboard to share with the group. We have hosted Ideation Jams with participants, including faculty, staff, and community partners, that bring a diverse range of expertise and experience. While we will always gravitate toward an in-person experience, we have delivered many successful Ideation Jams in a virtual format, which are a great option when geographic distance presents a barrier to collaboration.

More information about Research Jams can be found here:

- [Mobilizing cross-disciplinary teams to advance translational research using design thinking methods](https://web.archive.org/web/20230206160704/https:/www.cambridge.org/core/services/aop-cambridge-core/content/view/0096117CFD1F99BB7829B9C67103A666/S2059866121008232a.pdf/mobilizing_crossdisciplinary_teams_to_advance_translational_research_using_design_thinking_methods.pdf)
- [Collective creativity: strategies for catalyzing interdisciplinary research](https://web.archive.org/web/20230206160704/https:/jcom.sissa.it/archive/19/04/JCOM_1904_2020_C01/JCOM_1904_2020_C05)
- [Research Jams](https://web.archive.org/web/20230206160704/https:/michr.umich.edu/rdc/researchjams)

INTERVENTION DELIVERY: For CTSAs that have research groups that may benefit from an Ideation Jam, we can offer step-by-step support in its design and delivery. We will work with the point of contact for intervention delivery to 1) build a process timeline, 2) meet with the ‘faculty champion(s)’ of the effort to understand short- and long-term goals, 3) refine the Ideation Jam framework based on needs and desired outcomes of the faculty champion, 4) walk through all steps of the final Ideation Jam framework, 5) co-facilitate the first session, and 6) help with post-session activities (distribution of survey, creation of report, debrief with the faculty champion). We also have a large library of resources we can share, including process documents, timelines, recommendations for visuals, email communications, report templates, supply lists, participant surveys, whiteboard templates for virtual sessions, etc. Following delivery of the first Ideation Jam, our team can be available for guidance and advice with additional sessions moving forward; with enough interest, we would also be open to building a community of practice for ongoing learning and discussion.

**4. TeamMAPPS [8,9]**

**Project Leader:** Dr. Kevin Wooten

**Project Institution:** University of Texas Medical Branch

**Module Description:**

*What Is It?*

- **TeamMAPPS (Team Methods to Advance Processes and Performance in Science)**is a behavioral skills training program specifically developed to increase the collaboration and effectiveness of scientific teams and scientific team members.

*Who Developed TeamMAPPS?*

- Funded by the **University of Texas Medical Branch at Galveston**, well-published team science scholars (Dr. Eduardo Salas, Dr. Maritza Salazar, Dr. Theresa Lant, and Dr. Kevin Wooten) developed TeamMAPPS based on available evidence surrounding team effectiveness. The program was developed specifically for scientists and students to develop behavioral skills by learning mastery steps through Team Science cases and exercises, self-assessments, reflective exercises, and back-home application planning.

*What Does It Cover?*

- **TeamMAPPS** currently has five specific modules, including an Introductory Module (Welcome, Overview to Team Science, and Pre-Assessment Scales and Exercises), three Core Modules (below), and a Synthesis Module (Summary of Course, Reflective Exercises, and Implementation Planning). Core Modules include:
  - **Awareness and Exchange** (Sharing Unique Information/Promotive Voice, Inquiring/Probing, Reframing and Integrating)
  - **Psychological Safety** (Perspective Seeking, Acknowledgement and Inclusion, Addressing Issues and Resolving Conflict)
  - **Adaptation and Correction** (Monitoring and Debriefing, Reflecting and Analyzing, Creating Change/Development Plans)

*How Delivered/Facilitated?*

- TeamMAPPS can be delivered in three modalities across learner groups and teams differing in maturation:
  - **Completely Online/Individual:**web-based asynchronous delivery (across all learner groups)
  - **Face to Face Classroom/Seminar:** web-assisted using all modules or selected modules (e.g., Team Science workshop across all learner groups, graduate class, trainee or scholar seminar)
  - **Whole Team Intervention:** web-assisted using all modules or select modules for nascent or experienced scientific teams (e.g., study/proposal planning, team startup and development, change intervention)

**Future Modules**

- TeamMAPPS will be providing future developments including:
  - Smartphone-Based Personal App
  - Integrating Community Members Module
  - Integrating Commercialization Members Module

**5. Toolbox Dialogue Initiative [10,11]**

**Project Leader:** Dr. Marisa Rinkus

**Project Institution:** Michigan State University

**Module Description:**

Developed by the Toolbox Dialogue Initiative (TDI), the Toolbox dialogue method is an evidence-informed facilitation approach that surfaces implicit assumptions and diverse perspectives in complex, cross-disciplinary research projects for joint consideration and coordination. TDI has facilitated this process with more than 475 research teams and groups around the world, including work with the National Science Foundation, NASA, and multiple universities. For additional information about TDI see our website [https://tdi.msu.edu](https://web.archive.org/web/20230206160704/https:/tdi.msu.edu/)

*What is a Toolbox Dialogue?*

A Toolbox dialogue is a capacity building workshop that consists of an opening preamble to prepare the participants for the experience, followed by a 60 to 90-minute structured dialogue using a survey-like instrument called the “Toolbox” and ending with a co-creation activity designed to leverage what is learned in the dialogue. The Toolbox instrument uses a series of statements or prompts to structure the dialogue by providing topics for discussion and inviting reaction using a 5-point response scale (disagree to agree), along with don’t know and not applicable choices, as would be found in a survey. The prompts are designed to stimulate dialogue, a joint activity involving interaction among participants that requires active engagement through listening, reflecting, exploring, elaborating, analyzing, questioning, negotiating, and feedback-seeking.

Participation in a Toolbox dialogue facilitates reflection and perspective taking behavior in teams enabling collaborators to enhance their mutual understanding, specifically about the often-implicit beliefs, values, and priorities that structure their contributions to research. In doing this, a Toolbox dialogue supports the creation of a culture of communication and collaboration within the team. The dialogue is then leveraged through a co-creation activity to produce a deliverable (e.g., project concept map, project glossary, collaboration plan, success metrics) that the teams can use throughout their collaboration.

*Toolbox Dialogue Method Training for CTSA Hub Leaders*

This 3-part training is designed to enable CTSA Hub leaders in facilitating Toolbox-style dialogue with research teams. Parts I and II will provide a theoretical, experiential, and practical introduction to the Toolbox Dialogue Method, with a focus on designing and facilitating Toolbox-style dialogue. Part III will involve follow-up coaching and be designed with input from participants to further develop their capacity to design and implement Toolbox-style dialogues, address challenges faced or anticipated, and provide instruction on how to use Toolbox dialogues to facilitate other interventions (e.g. collaboration planning).
All sessions will include time for participant introductions and networking, discussion and Q&A, and breaks. Participants will also receive reference materials from the training including a handout summarizing all content, presentation slides, sample Toolbox instruments, and other resources. Training elements will occur virtually using the Michigan State University Zoom Pro License. Participants are expected to have a stable internet connection that will allow for use of video, particularly during the dialogue portions of the workshop. This workshop will be highly interactive and require a stable internet connection for audio and video use.

*Workshop Costs*

Virtual Toolbox Dialogue Method Training Workshop Parts I, II & III (9 hours): $1,500 per participant

**References**

1. Weiner BJ, Lewis CC, Stanick C, et al. Psychometric assessment of three newly developed implementation outcome measures. *Implementation Science*. 2017/08/29 2017;12(1):108. doi:10.1186/s13012-017-0635-3

2. Gallavin G. System Usability Scale (SUS): Improving Products Since 1986. Accessed July 24, 2024. <https://digital.gov/2014/08/29/system-usability-scale-improving-products-since-1986/>

3. Rolland B, Scholl L, Suryanarayanan S, et al. Operationalization, implementation, and evaluation of Collaboration Planning: A pilot interventional study of nascent translational teams. *J Clin Transl Sci*. Jul 24 2020;5(1):e23. doi:10.1017/cts.2020.515

4. Rolland B, Venkatesh S, Brasier AR. Optimizing an evidence-based team-building intervention for dissemination: Collaboration Planning 2.0. *Journal of Clinical and Translational Science*. 2025;9(1):e234. e234. doi:10.1017/cts.2025.10161

5. McCormack WT, Levites Strekalova YA. CTS teams: a new model for translational team training and team science intervention. *J Clin Transl Sci*. 2021;5(1):e183. doi:10.1017/cts.2021.854

6. LaPensee E, Salem B, Bugden J, et al. 559 Ideation Jams: Catalyzing Interdisciplinary Teams to Maximize Research Impact. *Journal of Clinical and Translational Science*. 2024;8(s1):167-167. doi:10.1017/cts.2024.476

7. LaPensee E, Doshi A, Salem B, et al. Mobilizing cross-disciplinary teams to advance translational research using design thinking methods. *Journal of Clinical and Translational Science*. 2021;5(1):e184. e184. doi:10.1017/cts.2021.823

8. Bisbey TM, Wooten KC, Salazar Campo M, Lant TK, Salas E. Implementing an evidence-based competency model for science team training and evaluation: TeamMAPPS. *J Clin Transl Sci*. 2021;5(1):e142. doi:10.1017/cts.2021.795

9. Molldrem S, Luft H, Farroni JS, Lyons EJ, Wooten K. Implementing TeamMAPPS: Formative qualitative findings from the dissemination and implementation study of a new evidence-based team science intervention. *Journal of Clinical and Translational Science*. 2025;9(1):e68. e68. doi:10.1017/cts.2025.22

10. Rinkus MA, Donovan SM, Hall TE, O’Rourke M. Using a survey to initiate and sustain productive group dialogue in focus groups. *International Journal of Social Research Methodology*. 2021/05/04 2021;24(3):327-340. doi:10.1080/13645579.2020.1786240

11. The Toolbox Dialogue Initiative: The Power of Cross-Disciplinary Practice. CRC Press; 2020.
